# Positive parenting: a randomised controlled trial evaluation of the Parents Plus Adolescent Programme in schools

**DOI:** 10.1186/s13034-015-0077-0

**Published:** 2015-08-25

**Authors:** Eileen Nitsch, Geraldine Hannon, Eóin Rickard, Sharon Houghton, John Sharry

**Affiliations:** University of Limerick, Limerick, Ireland; Parents Plus Charity, ℅ Mater Hospital, 15 St Vincent Street North, Dublin 7, Ireland

## Abstract

**Background:**

The aim of this study was to evaluate the Parents Plus Adolescents Programme (PPAP)—a parent training course specifically targeting parents of young adolescents (aged 11–16 years)—when delivered as a preventative programme in community school settings.

**Methods:**

A sample of 126 parents (mean age of children = 12.34 years; range = 10–16 years) were randomly assigned to either a treatment (PPAP; *n* = 82) or a waiting-list control condition (WC; *n* = 44). Analyses are based on a study-completer sample post-treatment (n = 109 parents: PPAP n = 70; WC n = 39) and sample at 6 month follow up (n = 42 parents).

**Results:**

Both post-treatment (between groups) and 6-month follow-up comparisons of study completers (within PPAP group) revealed significant positive effects of the parenting intervention with respect to adolescent behaviour problems and parenting stress. The post treatment comparisons demonstrated large effect sizes on global measures of child difficulties (partial eta squared = 0.15) and self-reported parent stress (partial eta squared = 0.22); there was a moderate effect size on the self-reported parent satisfaction (partial eta squared = 0.13).

**Conclusions:**

This study provides preliminary evidence that PPAP may be an effective model of parent-training implemented in a community-based setting. The strengths and limitations of the study are discussed.

## Background

Antisocial behaviour in young people is a growing problem. In the US and UK, 5–10 % of children aged 5–15 years present with clinically significant conduct disorders [1], while adolescent risk behaviour is deemed a persistent problem and a significant cause of youth morbidity and mortality [2]. Behaviour problems in adolescence are costly, due to the trauma and psychological problems caused to others who are victims of crime, aggression, or bullying, together with the financial costs to services for treatment of both the condition and its long-term sequelae. Use of health, social, education, and legal services is ten times higher for this population, and this usage is mostly borne by publicly funded services, especially in areas of social exclusion [3]. A UK study conducted by Scott, Knapp, Henderson, and Maughan [4] suggested that, by age 28 years, the costs of individuals with a clinical diagnosis of conduct disorder were ten times higher than for those with no problems, and costs for those with conduct problems not meeting the diagnostic criteria were 3½ times higher.

Overwhelmingly, the research literature confirms the strong and enduring influence of parenting practices on adolescents [5]. Research has shown that poor parenting skills (e.g., harsh, authoritarian, disproportionately punitive, laissez-faire; [6, 7] and inconsistent parenting strategies [8] can lead to undesirable outcomes in children and adolescents. These outcomes include behavioural and emotional problems [9], externalising and internalising behaviours [7], and decreased cognitive and academic development [10]. Additionally, poor parenting skills have been linked with poorer self-regulation in children and adolescents [11], use of aggression [8], and severe behavioural problems that persist over time [12].

### Parent training and adolescence

Internationally, the majority of parenting interventions target pre-adolescent children [13, 14] with a smaller number targeting the needs of adolescents (e.g., the Triple P Teen programme [15] and the Strengthening Families Programme [16]). Generally, parenting education is viewed as a valuable way of avoiding adolescent anti-social behaviour and, therefore, more costly interventions in the future [17]. Indeed, there is a body of research suggesting that early intervention with parents (e.g., starting in infancy or early childhood) can be effective in reducing adolescent behaviour problems [18].

There are, however, several reasons for developing and utilising adolescent-specific parenting programmes. Not all families successfully engage in parenting programmes when children are younger, and only seek help during their adolescence. Even parents who have attended previous courses may need to re-attend during adolescence to tackle the emergence of new problems. Moreover, adolescence brings with it a range of new developmental and social challenges that are not present or relevant when children are young (e.g., alcohol use). Discipline strategies will also necessarily change during the adolescent period, with strategies that were effective with younger children no longer effective or developmentally desirable. Certainly, research has shown that for parenting programmes to be effective they must be developmentally timed to be relevant to the parent’s needs [19].

Given the evidence confirming the strong and enduring influences of parenting practices on adolescents, there is a great need for increased accessibility to community-based parent-training programmes targeted at the needs of adolescents. In addition, corresponding evaluations of these programmes need to be conducted to determine their effectiveness, given the potential challenges of this age group and the possibility that problems may be more fixed and less amenable to change.

In relation to the delivery of such a programme, schools have been identified as a natural, suitable, and, in some cases, preferred location for the provision of mental health services [20], with the benefits of basing preventative and intervention programmes in the school setting being well-established (e.g., Lean and Corlucci [21], Van Acker and Mayer [22]). In the US, for example, it has been reported that 75 % of children and adolescents who receive mental health services do so through their schools [23].

Furthermore, it has frequently been reported that the period during and after the transition from primary to post-primary school can be extremely challenging for young people [24], with a reported increase in behavioural [25], academic [26], and discipline problems [27] accompanying the change. Factors such as the onset of puberty [24], concern about knowing the new rules/procedures of the school [28], and secondary school being a more intimidating environment [29] have all being identified as stressors during this period. Given the apparent increased likelihood of difficulties occurring in the transition to secondary school, it was decided to specifically target this time period in the current research, with schools being chosen as the location for the roll-out of the Parents Plus Adolescents Programme (PPAP), as an extension of previous research evaluations of the PPAP.

### Parents Plus Adolescents Programme

The PPAP [30] is a group-based training intervention for parents of young adolescents aged 11–16 years. It is one of three Parents Plus Programmes targeting different age groups, with corresponding programmes for primary school [31] and preschool children [32].

Like international programmes such as Triple P Teen [15] and Strengthening Families [16], the PPAP draws largely from a social learning theoretical background, but also incorporates ideas from conflict management and negotiation models [33] and discipline strategies from Parent Effectiveness Training [34]. The PPAP differs from the international programmes in that the programme materials and DVD footage was developed with Irish parents and teenagers and the delivery of the programme draws from a solution-focused and strengths-based collaborative approach to working with families [35, 36]. In addition the PPAP facilitators use parent evaluations of sessions, which are not anonymised, to tailor the delivery of the programme to the specific group of parents attending and to be proactive in guarding against attrition by speaking with parents who are not satisfied with the programme.

The PPAP includes two DVDs, a facilitator’s manual, and an accompanying parent handbook. The manual contains extensive background information, a guide on how to prepare and run each session, and hand-outs and home-work assignments for participants. The DVDs contain 2 h footage of both acted and real scenes of parenting situations.

### Programme content

The central philosophy in each group session is balance. The aim each week is to introduce one positive parenting idea (e.g., listening) and one discipline/behaviour management idea (e.g., using consequences), giving parents two new ideas to reflect on and practice. This makes the course positive and preventative, while also tackling the behaviour problems that parents are concerned about. An outline of the topics covered over the 8 weeks is seen in Table 1. While some of the topics covered in PPAP are similar to the original programme aimed at parents of younger children (e.g., social learning principles), there are also a number of differences. For example, in the PPAP the skills of connecting, relationship building and problem solving covered in Sessions 1–7 are specific to an adolescent’s stage of development who is in the process of becoming independent from the family.

**Table 1:** Overview of PPAP course content

Session	Content
Session 1	Introduction to course
	Positive communication
Session 2	Getting to know your teenager
	Communicating rules positively
Session 3	Connecting with your teenager
	Communicating rules positively
Session 4	Encouraging your teenager
	Using consequences
Session 5	Listening to your teenager
	Having a discipline plan
Session 6	Empowering teenagers
	Dealing with conflict and aggression
Session 7	Problem solving with young people
	Dealing with specific issues
Session 8	Dealing with specific issues
	Parental self-care
	Closing and course evaluation

### Parents Plus research basis and the evidence-base for parent management training in adolescence

There are currently 12 published studies providing evidence for the effectiveness of the Parents Plus Programmes in reducing behavioural problems and parental stress in a variety of contexts and with a variety of age groups (e.g., [37–43]).

For example, in a large-scale, multisite controlled study of children aged 1–6 years (*N* = 97), findings indicated that parents completing the Parents Plus programme reported significant decreases in child problem behaviour and parental stress, a reduction in commands, and an increase in positive attends in the parent–child interaction post-intervention [39]. Additionally, no significant difference in benefit was identified between children with developmental delays and children with primarily behaviour problems, suggesting that the PPEY may be equally beneficial to both groups and could be used as broad-based intervention in child mental health services [39].

Though there are less studies, research evidence supports the efficacy of parent management training in adolescence. In particular, there is a growing body of evidence for the effectiveness of international Parenting Programmes targeting teens such as Teen Triple P [44, 45] and the Strengthening Families Programme [16, 46].

There has been one previous small controlled evaluation of the PPAP in an adolescent mental health setting with 55 families indicated that, compared to wait-list controls, parents completing the PPAP reported higher goal attainment, greater improvements in their relationship with their child, and a reduction in behavioural difficulties (i.e., improvements on the SDQ Total Difficulties scale, and Peer and Conduct subscales) when compared to waiting-list control group [47].

The overall aim of the current study is to expand upon this research by examining the effectiveness of the Parents Plus Adolescents Programme as a preventive programme for parents of children in the process of transition to second level schooling. The primary outcome expected was that there would be a reduction in any child emotional and behaviour problems, which would be both statistically and clinically significant. Positive outcomes for parents in terms of increased parents satisfaction and reduced parental stress are also expected after attendance at the eight-week programme and subsequent improvements are expected to be maintained at six-month follow-up. We also included a parent-report measure to evaluate parent’s attainment of personal goals and parent-defined goals for their child identified prior to starting the programme.

## Methods

### Study design

**Fig. 1:**
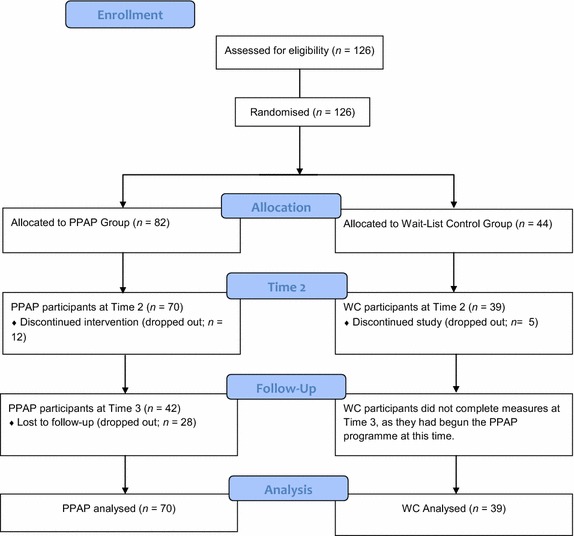
PPAP RCT participant flow

This study utilised a Randomised Controlled Trial (RCT) design, in which 126 parents were randomly assigned to one of two conditions: PPAP or a waiting-list control group. The waiting-list control group did not receive any intervention during the wait period and were enrolled in the subsequent PPAP programme. Assessments were conducted prior to programme delivery (Time 1), immediately after programme delivery (Time 2), and at six-month follow-up (Time 3). The waiting-list control group completed assessments at the pre- and post-assessment stages only (Fig. 1).

### Procedure

The study received ethical approval from the Research Ethics Committee of the University of Limerick, Ireland. The study was undertaken from September 2009 to September 2010. The PPAP was delivered in primary and post-primary schools throughout counties Cork and Kerry in the Republic of Ireland. As part of a county-wide initiative to support adolescents transitioning from primary to post-primary schools, families with children in the last year of primary school and the first year of secondary school were particularly targeted. Schools whose principals expressed an interest were selected for participation, dependent on the availability of locally trained community facilitators. Participant enrolment was conducted by the staff at each school.

In order to recruit parents, promotional materials were provided to participating schools and information letters sent to all parents. The PPAP information evenings were also advertised in local communities via notices in local newspapers and newsletters. As the programme was implemented as a preventative group, an open recruitment strategy was used, and the only mandatory inclusion criterion was that the child of concern to the parent was between 10 and 16 years. Sample size was thus determined by the number of parents who were willing and able to take part.

An information evening was held at participating schools where the PPAP was introduced and explained. Parents who expressed an interest in attending the parenting programme were also invited to take part in the study and those who agreed completed a consent form and the set of standardised assessment measures. The inclusion criterion for was that children were aged 10–16 years; there were no specific exclusion criteria. At the end of the information session the primary researcher then randomly allocated participating parents to either the PPAP group or WC group by assigning sequentially numbered envelopes, with those allocated to the WC condition made aware that they would be participating in the subsequent PPAP group. Randomisation was done on a 2:1 basis. The rationale for choosing a 2:1 ratio was to increase the number of treatment groups and thus increase the study’s power for a fixed total sample size. In addition, a high drop-out rate was anticipated. As this study was a waiting-list controlled RCT, participants, care providers, and those assessing outcomes were aware of which experimental group they had been assigned to after random allocation.

### PPAP and programme facilitators

All participants in the treatment group attended one of ten Parents Plus Adolescent Programmes (PPAP) thus receiving the same 8 week video-modelling parenting intervention. Each group was delivered by two facilitators. The main facilitator for each group was experienced in delivering the PPAP, and co-facilitators for each group underwent an intensive 2-day facilitators training course, delivered by the programme authors, prior to programme implementation. All facilitators had a professional background in health or education. Treatment fidelity and integrity was adhered to via the use of a manualised programme. Facilitators completed adherence checklists at the end of each group session, and also attended a small group supervision sessions with other facilitators during the group delivery.

### Participants and attrition

Participants recruited at baseline were 126 parents who were randomly assigned to either the PPAP group (*n* = 82) or the Waiting-list Control (WC) group (*n* = 44). Only one parent per family participated in the study. Parents who attended five or more of the eight parenting sessions were included in the final analyses. Of the 126 parents, a total of n = 109 (86.5 %) completed Time 2 measurements. This figure included 85.4 % (n = 70) of the PPAP Group and 88.6 % (n = 39) of the WC group. The remaining 13.5 % (n = 12) of participants in the PPAP Group did not complete Time 2 measurements because they dropped out of the PPAP. The remaining 13.6 % (n = 5) of the WC group also dropped out of the study. Data from dropouts were excluded from the final analysis.

The young people of concern to the parents who completed the PPAP had a mean age of 12.34 years (SD = 1.36; range 10–16 years) and the majority were female (61 %, n = 66 vs. Male: 39 %, n = 43). In the PPAP Group, 10 % of the children were receiving a clinical service compared with 8 % in the WC group. These clinical services included Occupational Therapy, Speech and Language Therapy, Psychology and Cardiology. Statistical analysis demonstrated no significant difference between the groups based on this variable. The demographic characteristics of the parents and their children in the PPAP group and the WC groups are presented in Table 2.

**Table 2:** Demographic information

Variable	PPAP group (*n* = 70)		WC group (*n* = 39)	
	*N*	%	*N*	%
Parents				
Parent type^a^				
Mother	61	87	32	82
Father	9	13	6	15
Foster Mother	–	–	1	3
Mothers’ employment status				
Public sector	22	36	16	50
Private sector	6	10	–	–
Health service	3	5	6	19
Full-time homemaker	28	46	11	31
Student	2	3	–	–
Fathers’ employment status				
Public sector	7	78	2	34
Full-time homemaker	2	22	2	33
Health service	–	–	2	33
Children				
Gender				
Male	27	39	16	41
Female	43	61	23	59
Receiving a Clinical Service (educational, speech and language, psychological, etc.)				
Yes	7	7	3	8
No	63	63	36	92

^a^Only one parent per family participated in the study

The intervention group was followed up after 6 months. The attrition rate from the PPAP Group at 6 month follow up was 49 % (n = 40 parents), with n = 42 parents providing follow up data. Descriptive data indicate that the parents (n = 28) who dropped out between Time 2 and Time 3 (6-month follow up) obtained higher mean Total Difficulties Score on the SDQ at Time 2 compared with parents (n = 42) who completed outcome measures at Time 3. These parents (n = 28), who were lost to follow up at Time 3, also reported lower levels of satisfaction with the parenting role at Time 2, although there was only a one point difference between the groups on the self-report satisfaction measure; they also reported lower levels of parenting stress compared with study completers. Of note is that these differences reported on post intervention measures were not statistically significant.

### Instruments

#### Demographic Questionnaire

The Demographic Questionnaire was developed specifically for the current evaluation study. This instrument was designed to gather family demographic information including contact details, marital and employment status.

#### Strengths and Difficulties Questionnaire

*(SDQ)* The SDQ [48] is a 25-item behavioural screening inventory, which asks parents about pro-social and difficult behaviour in children aged 4–16 years. It consists of five subscales (Emotional Problems; Conduct Problems; Hyperactivity; Peer Problems; and Prosocial behaviour), each with five items. A Total Difficulties Score is derived from the combined scores of the first four scales, and a score of 17 or above is in the ‘abnormal’ range. The subscales have a mean internal consistency reliability coefficient of 0.71, mean test–retest reliability co-efficient over 6 months of 0.62, and strong criterion validity for predicting psychological disorders [49]. The Cronbach’s alpha coefficient in the current study was 0.81 for the Total Difficulties scale, and >0.7 for the Hyperactivity, Emotional Problems, and Prosocial subscales. The reliability coefficient for the Conduct Problems subscale was 0.51, while Peer Problems yielded an alpha of 0.69.

#### Parenting Stress Index–Short Form (PSI/SF)

This measure is a direct derivative of the full-length (120 item) test and consists of a 36-item parent report scale, with a 5-point Likert response format [50]. The scale yields a total parenting stress score on three 12-item subscales: Parental Distress; Difficult Child; and Parent–Child Dysfunctional Interaction. As the majority of children of participants (74 %) in the current study were aged 12 years or under, the 0–12 years version of the PSI/SF was used. The total scale and subscales of the PSI/SF have been found to have internal consistency reliability coefficients above 0.9 and test–retest reliability coefficients of between 0.65 and 0.96 [51]. The alpha coefficient in the current study was 0.88 for the Total Parenting Stress subscale and 0.81 for the Parental Distress and Difficult Child subscales, while the Parent–Child Dysfunctional Interaction subscale yielded an alpha value of 0.77.

#### Kansas Parenting Satisfaction Scale (KPSS)

This is a 3-item instrument designed to measure an individual’s satisfaction with themselves as a parent, the behaviour of their children, and their relationship with their children [52]. A 7-point Likert response scale is used, and the scores for all three items are summed to yield a total parenting satisfaction score. The KPSS has been found to have internal consistency reliability coefficients ranging from 0.78 to 0.95 [53]. The alpha coefficient for the KPSS in the current study was 0.82.

#### Parents Plus Goal Form

The Parents Plus Goal Form was designed by the authors of the Parents Plus Programmes in order to (1) evaluate attainment of target goals that parents identify in relation to their child; and (2) evaluate parent’s attainment of personal goals identified prior to starting the programme. A visual analogue scale was used to measure parents’ rating of goal attainment, and they listed two goals for themselves and two for their child. Parents indicated how far they and their child were, at the time of completion, from achieving each goal by rating it from 0 to 10 on the analogue scale, where 0 is ‘very far away from goal’ and 10 is ‘have reached this goal’. A mean goal score is calculated, again ranging from 0 to 10. At subsequent data collection times (each week and at Time 2 and Time 3 data collection) parents reviewed their initial goals and indicated how close they and their child now were to achieving their goals. Examples of common goals reported by parents on the PPGF can be seen in Table 3.

**Table 3:** Examples of goals stated by parents on the PPGF at pre-intervention

Goals for their child	Goals for themselves
To confide more in their parents	To have better awareness of possible conflict
To be more independent	To stop losing my temper so quickly
To understand and accept boundaries	To be able to listen more
To have a more positive relationship with us	To be less critical and more accepting of my child
To communicate problems better	To better deal with conflict
To have more confidence in themselves	To be more patient

### Analyses

Data were analysed using the PASW Statistics 18 package for Windows. Prior to analyses all variables were examined for accuracy of data entry, missing values, presence of outliers, and normality of distribution. Inspection of the distribution of scores on the continuous dependent variables showed that the scores were reasonably normally distributed.

Descriptive statistics: namely; percentages, means, frequencies and standard deviations were computed for demographic variables. We also tested for differences between the PPAP and WC groups on parent and child demographic characteristics and Time 1 scores on the standardised outcome measures using independent samples t tests and Chi square tests. In order to reduce the risk of obtaining a Type 1 error, a more stringent alpha value was set at 0.003 (Bonferroni adjustment).

One-way repeated measures analysis of variance (ANOVA) was used to examine change over time (Time 1–3) for the intervention group on standardized measures. Where significant differences were found in the one-way ANOVAs across the three time stages, paired-samples t-tests were employed in order to illuminate the nature of observed differences in mean scores.

A series of mixed ANOVAs were used to test for interactions between time (Time 1 and Time 2) and group (PPAP and WC) on the same standardized measures. Where significant interactions were observed tests of simple effects were carried out to further examine the nature of the interactions. Effect sizes were evaluated in accordance with Cohen’s (1998) guidelines: 0.01 = Small effect, 0.06 = Moderate effect, 0.14 = Large effect.

## Results

### Preliminary analyses

Results of independent samples t tests and Chi squared analyses on parent and child demographic characteristics at study entry showed no significant differences between the PPAP and WC groups at study entry for any variable.

Independent-samples t tests also revealed no significant difference in scores between the two groups for any of the measures administered to participants at Time 1 (see Table 4), with the exception of the Emotional Symptoms subscale of the SDQ, where the mean score for the PPAP group (*M* = 3.93, SD = 2.41) was found to be significantly higher than the WC group (*M* = 2.56, SD = 2.10). As the overall Total Difficulties score for the SDQ did not significantly differ between the groups, this aspect is not critical, but findings for this individual subscale should be interpreted with caution.

**Table 4:** Independent t test results for all assessments administered at Time 1 (*N* = 109)

Scale	*t*	*p*	*η* ^2^
SDQ			
TD	*t* (107) = 0.92	0.360	0.008
HYP	_^a^		
CON	*t* (107) = -0.53	0.596	0.003
EMOT	*t* (107) = 2.96	0.004*	0.075
PEER	*t* (107) = 1.01	0.317	0.009
PRO	*t* (107) = 0.65	0.515	0.004
PSI			
PSI total	*t* (107) = 1.99	0.054	0.036
PD	*t* (107) = −0.85	0.399	0.007
DC	*t* (107) = 1.15	0.253	0.012
CDI	*t* (107) = 0.71	0.479	0.005
Parent-Defined Goals			
PDPG	*t* (107) = −0.88	0.379	0.007
PDCG	*t* (107) = 1.07	0.287	0.010
KPSS			
KPSS total	t (107) = 0.798	0.427	0.006

*SDQ* Strengths and Difficulties Questionnaire, *TD* Total Difficulties, *EMOT* Emotional Symptoms, *HYPER* Hyperactivity, *CON* Conduct Problems, *PEER* Peer Problems, *PRO* prosocial behavior, *PSI* Parenting Stress Index, *PSI total* total stress, *PD* parental distress, *DC* Difficult Child, *P-CDI* Parent–Child dysfunctional interaction, *KPSS* Kansas Parenting Satisfaction Scale, *KPSS total* KPSS total parenting satisfaction, *PDCG* Parent-Defined Child Goals, *PDPG* Parent-Defined Personal Goals

* *p* < 0.05

^a^Given that no higher order effects were observed for the SDQ-Hyperactivity subscale the main effects detected for them can be reported. No main effect for group was observed for this subscale

### Comparison of means of assessment measures for the PPAP and WC groups

Treatment effects observed for the PPAP group were compared with the WC group. Because there was no data collected for the WC group at Time 3, only Time 1 and Time 2 data were considered. In order to explore for Group × Time interactions, Time effects, and Group effects, each dependent variable was analysed using a two-way ANOVA.

### ANOVA results for SDQ, PSI/SF, KPSS, and PDGF

Change over time for the PPAP and WC groups was examined across the dependent measures, SDQ, PSI/SF, KPSS, and PDGF. The means and standard deviations for the PPAP and WC groups at Time 1 and Time 2 for each of the standardised scales are presented in Table 5.

**Table 5:** Mean scores for PPAP and WC groups on SDQ, PSI, and KPSS scales across Time 1 and Time 2 (standard deviations in parentheses)

Scale	PPAP group (n = 70)		WC group (n = 39)	
	Time 1	Time 2	Time 1	Time 2
SDQ				
TD	11.97 (5.96)	5.70 (4.13)	10.82 (6.78)	10.92 (6.80)
HYP	3.33 (2.28)	2.86 (1.84)	3.79 (2.76)	3.90 (2.69)
CON	2.30 (1.61)	0.886 (1.09)	2.49 (2.01)	2.38 (2.16)
EMO	3.93 (2.41)	0.986 (1.62)	2.5 (2.10)	2.56 (1.94)
PEER	2.41 (2.26)	0.971 (1.44)	1.97 (2.07)	2.08 (1.91)
PRO	6.90 (2.53)	8.33 (1.63)	7.21 (1.94)	7.38 (1.94)
PSI				
PSI total	77.97 (14.69)	65.87 (14.39)	84.10 (18.13)	83.95 (19.44)
PD	28.17 (7.65)	21.59 (6.36)	29.46 (7.57)	28.97 (8.45)
DC	30.71 (7.66)	23.21 (6.25)	28.95 (7.71)	29.41 (7.96)
PSI-P-CDI	26.16 (6.34)	21.07 (5.01)	25.23 (6.85)	25.56 (6.84)
KPSS				
KPSS total	14.23 (16.89)	16.89 (2.36)	13.72 (2.88)	13.62 (3.08)
Parent-Defined Goals				
PDCG	3.97 (1.54)	6.53 (1.42)	4.31 (1.62)	4.15 (1.66)
PDPG	3.63 (1.48)	6.93 (1.38)	3.90 (1.60)	3.87 (1.66)

*SDQ* Strengths and Difficulties Questionnaire, *TD* Total Difficulties, *EMOT* Emotional Symptoms, *HYPER* Hyperactivity, *CON* Conduct Problems, *PEER* Peer Problems, *PRO* prosocial behavior, *PSI* Parenting Stress Index, *PSI total* total stress, *PD* parental distress, *DC* Difficult Child, *P-CDI* Parent–Child dysfunctional interaction, *KPSS* Kansas Parenting Satisfaction Scale, *KPSS total* KPSS total parenting satisfaction, *PDCG* Parent-Defined Child Goals, *PDPG* Parent-Defined Personal Goals

Significant interaction effects were observed across parents’ mean scores for the Total Difficulties score and all subscales, with the exception of SDQ-Hyperactivity, and for parent-defined goals (see Table 6).

**Table 6:** Mixed between-within ANOVA group, time, and interaction main effects for SDQ, PSI, and KPSS scales across the PPAP and WC groups

Scale	Group		Time		Interaction	
	*F*	*p*	*F*	*p*	*F*	*p*
SDQ						
TD	*F* (1, 107) = 3.53	0.063	*F* (1, 107) = 60.01	<0.001***	*F* (1, 107) = 64.07	<0.001***
HYP	*F* (1, 107) = 3.28	0.073	*F* (1, 107) = 0.787	0.377	*F* (1, 107) = 1.91	0.170
CON	*F* (1, 107) = 7.98	0.006*	*F* (1, 107) = 26.74	<0.001***	*F* (1, 107) = 19.98	<0.001***
EMOT	*F* (1, 107) = 190.8	0.769	*F* (1, 107) = 62.76	<0.001***	*F* (1, 107) = 62.76	<0.001***
PEER	*F* (1, 107) = 0.895	0.346	*F* (1, 107) = 18.04	<0.001***	*F* (1, 107) = 23.98	<0.001***
PRO	*F* (1, 107) = 0.80	0.373	*F* (1, 107) = 15.23	< 0.001***	*F* (1, 107) = 9.19	<0.001***
PSI						
PSI total	*F* (1, 107) = 15.29	<0.001***	*F* (1, 107) = 46.40	<0.001***	*F* (1, 107) = 44.08	<0.001***
PD	*F* (1, 107) = 9.87	<0.001***	*F* (1, 107) = 45.04	<0.001***	*F* (1, 107) = 33.48	<0.001***
DC	*F* (1, 107) = 2.78	0.098	*F* (1, 107) = 33.55	<0.001***	*F* (1, 107) = 42.93	<0.001***
CDI	*F* (1, 107) = 2.59	0.111	*F* (1, 107) = 20.53	<0.001***	*F* (1, 107) = 26.69	<0.001***
KPSS						
KPSS total	*F* (1, 107) = 11.96	<0.001***	*F* (1, 107) = 36.03	<0.001***	*F* (1, 107) = 42.05	<0.001***

*SDQ* Strengths and Difficulties Questionnaire, *TD* Total Difficulties, *EMOT* Emotional Symptoms, *HYPER* Hyperactivity, *CON* Conduct Problems, *PEER* Peer Problems, *PRO* prosocial behavior, *PSI* Parenting Stress Index, *PSI total* total stress, *PD* parental distress, *DC* Difficult Child, *P-CDI* Parent–Child dysfunctional interaction, *KPSS* Kansas Parenting Satisfaction Scale, *KPSS total* KPSS total parenting satisfaction

* Significant at *p* < 0.05, *** significant at p < 0.001

### Within-group outcomes for the PPAP Group

A series of one-way repeated measures ANOVAs were used to evaluate changes in scores on parent measures from Time 1 (pre-intervention) to Time 2 (post-intervention) and Time 3 (at 6-month follow-up) within the PPAP group. The means, standard deviations, and main effects for time are displayed in Table 7.

**Table 7:** Means, standard deviations (in parentheses), and repeated measures ANOVA time effects for PPAP Group

Scale	Time 1 (*n* = 70)	Time 2 (*n* = 70)	Time 3 (*n* = 42)	ANOVA	Partial *η* ^2^
SDQ					
TD	11.97 (5.96)	5.70 (4.13)	4.90 (3.28)	*F* (2, 41) = 35.57, *p* < 0.001***	0.63
EMOT	3.93 (2.41)	0.986 (1.62)	0.837 (1.09)	*F* (2, 41) = 31.47, *p* < 0.001***	0.61
HYPER	3.33 (2.28)	2.86 (1.84)	1.86 (1.61)	*F* (2, 41) = 5.64, *p* = 0.007*	0.22
CON	2.30 (1.61)	0.886 (1.09)	1.28 (1.22)	*F* (2, 41) = 17.53, *p* < 0.001***	0.46
PEER	2.41 (2.26)	0.971 (1.44)	0.930 (1.24)	*F* (2, 41) = 11.51, *p* < 0.001***	0.36
PRO	6.90 (2.53)	8.33 (1.63)	8.23 (1.70)	*F* (2, 41) = 2.88, *p* = 0.068	0.12
PSI					
PSI total	77.97 (14.69)	65.87 (14.39)	65.86 (16.92)	*F* (2, 41) = 34.44, *p* < 0.001***	0.62
PD	28.17 (7.65)	21.59 (6.36)	21.60 (6.68)	*F* (2, 41) = 27.48, *p* < 0.001***	0.58
DC	30.71 (7.66)	23.21 (6.25)	23.22 (7.24)	*F* (2, 41) = 18.86, *p* < 0.001***	0.55
P-CDI	26.26 (6.34)	21.07 (5.01)	21.02 (4.94)	*F* (2, 41) = 17.16, *p* < 0.001***	0.46
KPSS					
KPSS total	14.23 (3.37)	16.89 (2.36)	16.58 (2.74)	*F* (2, 41) = 20.09, *p* < 0.001***	0.50

*SDQ* Strengths and Difficulties Questionnaire, *TD* Total Difficulties, *EMOT* Emotional Symptoms, *HYPER* Hyperactivity, *CON* Conduct Problems, *PEER* Peer Problems, *PRO* prosocial behavior, *PSI* Parenting Stress Index, *PSI total* total stress, *PD* parental distress, *DC* Difficult Child, *P-CDI* Parent–Child dysfunctional interaction, *KPSS* Kansas Parenting Satisfaction Scale, *KPSS total* KPSS total parenting satisfaction

* Significant at *p* < 0.05, *** significant at p < 0.001

#### Strength and Difficulties Questionnaire (SDQ)

There were significant differences between the mean scores of the two groups at Time 2, *t* (107) = 4.37, *p* < 0.05, *η*^2^ = 0.15, with the mean scores of the PPAP significantly lower than those of the WC group (large effect size). There were similar findings for the Conduct Problems, *t* (107) = −4.05, *p* < 0.05, *η*^2^ = 0.13, and Emotional Symptoms subscales, *t* (107) = −4.54, *p* < 0.05, *η*^2^ = 0.16, with large effect sizes for both, and parents in the PPAP group also reported significantly lower rates of problem behaviour on these variables at Time 2 compared to Time 1. There was no significant change over time reported by parents in the control group on any of these subscales of the SDQ. Parents in both the PPAP and WC groups reported only minor changes in SDQ Hyperactivity scores from Time 1 to Time 2.

Furthermore, a significant difference with a moderate effect size was revealed between groups on the SDQ Prosocial subscale at Time 2, *t* (107) = 2.70, *p* < 0.05, *η*^2^ = 0.064, with parents in the PPAP group reporting a significant increase in prosocial behaviour on completion of the parenting programme.

There were significant time effects for SDQ Total Difficulties and the Emotional Symptoms, Hyperactivity, Conduct Problems, and Peer Problems subscales, with large effect sizes for these changes. Paired-samples t tests among Time 1, 2, and 3 scores, identified significant differences in the mean scores for Total Difficulties, Conduct problems, Hyperactivity, and Emotional Symptoms between Time 1 and Time 2, and these changes were maintained at Time 3.

### Clinical improvement

Cases were classified as clinically improved if they moved from the clinical and borderline ranges to the non-clinical range on the SDQ Total Difficulties scale. Of the 24 (34.3 %) children in the PPAP group who scored in the clinical/borderline range at Time 1, 21 (30 %) of these moved into the non-clinical range at Time 2. This number further decreased to just 1 (2.3 %) child remaining in the clinical range at Time 3. This positive trend in terms of clinical improvement from Time 1 to Time 2 was not observed for the control group.

### Parent satisfaction, parental stress and child problems

#### Parenting Stress Index (PSI/SF)

A significant difference was revealed between the mean scores of the two groups at Time 2 for Total Parenting Stress, *t* (107) = −5.51, *p* < 0.05, *η*^2^ = 0.22; Parental Distress, *t* (107) = 5.16, *p* < 0.05, *η*^2^ = 0.199; Difficult Child, *t* (107) = −4.49, *p* < 0.05, *η*^2^ = 0.16; and Parent–Child Dysfunctional Interaction, *t* (107) = −3.93, *p* < 0.05, *η*^2^ = 0.13. The effect size for the difference between groups was large for the former three variables, and moderate to large for Parent–Child Dysfunctional Interaction. In essence, parents in the PPAP group reported a significant decrease in parenting stress following attendance at the parenting programme compared to the control group.

There were significant time effects for Total Parenting Stress and Parental Distress, Difficult Child, and Parent–Child Dysfunctional Interaction subscales for the PPAP group, with large effects sizes for these changes. Paired-samples *t* test analyses indicated a significant improvement in scores for each variable from Time 1 to Time 2, which was maintained at six-month follow-up.

#### Kansas Parenting Satisfaction Scale

A significant difference in KPSS scores between the two groups was identified at Time 2, *t* (107) = −3.93, *p* < 0.05, *η*^2^ = 0.13, with a moderate to large effect size for this difference. A significant effect for time was observed for the PPAP group, with a large effect size, *t* (69) = −8.97, *p* < 0.05, *η*^2^ = 0.53, but not for the WC group, *t* (38) = 0.503, *p* > 0.05, *η*^2^ = 0.001, The PPAP group reported a significant increase in overall parenting satisfaction from Time 1 to Time 2.

The mean score of the PPAP group increased significantly from Time 1 to Time 2, and Time 1 to Time 3, with a small, non-significant decrease from Time 2 to Time 3. There was a large effect size for the change from Time 1 to Time 3. Additionally, the mean scores at post-intervention and follow-up were in the non-clinical range, above the clinical cut-off score of 15 for the KPSS.

### Changes in parent defined goals

#### Parent-defined goals

For Parent-Defined Child Goals (PDCG), a significant difference with a large effect size was found between the PPAP and WC groups at Time 2, *t* (107) = 7.86, *p* > 0.05, *η*^2^ = 0.37. Similarly, for Parent-Defined Personal Goals (PDPG), a significant difference with a large effect size was found at Time 2, *t* (107) = 10.32, p > 0.05, *η*^2^ = 0.49, between the PPAP and WC groups. There was an interaction effect for parents’ mean scores on the Parent Defined Parent Goals (*F* (1, 107) = 131.3, p < 0.05) and Parent Defined Child Goals (*F* (1, 107) = 81.95, p < 0.05). However the p-value was lower than the Bonferroni adjusted level of p > 0.003.

## Discussion

The findings of the evaluation study provide encouraging results for the parents who attended the PPAP and for their children (mean age 12.34 years) who were transitioning to secondary school, suggesting that the programme is effective in reducing behaviour problems. It is encouraging to find that the reported improvements were maintained at the six-month follow-up stage, as this suggests that the treatment effects observed are sustainable over time. Despite the high rate of dropout to Time 3 (49 %), analyses did not demonstrate a statistically significant difference between PPAP completers at Time 2 and those who participated in the follow up at Time 3. The implication of this finding is that all completers benefited from completing the PPAP.

The results are broadly consistent with comparable studies on international programmes such as Teen Triple P [44, 45] as well as studies on the other Parents Plus Programmes (e.g., [38–40]), which have shown the positive impact of the parenting programmes on a range of parent and child outcomes, including improved child emotional and behaviour problems. One caution with regards to these findings is that the PPAP reported a significantly higher score on the SDQ Emotional Subscale at Time 1. Therefore, we cannot make a conclusive comment on effect of the PPAP on internalising problems in this sample.

Another important finding is the beneficial outcome in terms of parents’ well-being and parental competencies following programme attendance. Parents reported a significant decrease in levels of parenting stress and a significant increase in levels of parenting satisfaction following course attendance. We can hypothesise about possible mechanism of change, e.g., by attending a group-based intervention and sharing their experiences may have led to reduced parent stress, increased satisfaction and improved child outcomes. Another hypothesis is that parents learned and implemented new parenting skills, which led to the positive study outcomes. As we did not measure changes in parenting practices, the latter is a tentative hypothesis, which could be address in future research.

In terms of goal attainment, parents in the PPAP group reported significant advancement towards attainment of parent-defined child and personal goals compared to the WC group. The most common child and personal goals, as defined by parents at baseline were in relation to adolescent behaviour (e.g., compliance and cooperation) and positive parenting strategies (e.g., to stay calm). Making a direct comparison between treatment gains for the PPAP group against a randomised control group provides evidence that strongly suggests that these positive outcome gains resulted from parents’ attendance at the parenting programme.

### Methodological strengths and limitations

#### Strengths

The current study meets the criteria for a rigorous design (i.e., adequate sample size, randomised control). Another positive aspect of the study is that it was conducted in a ‘real world’ setting.

The inclusion of a 6-month follow-up attests to the sustainability of the treatment effects and rules out change due to short-term fluctuations. Conducting a follow-up is important for establishing the social validity of an intervention [54]. Keeping with this issue of follow-up assessment, prolonging the period before the follow-up assessment to 1 year or conducting a second stage of follow-up would be necessary steps to further test the sustainability of treatment effects observed following the PPAP.

### Limitations

However, there are methodological limitations to be noted. A major limitation of this study is that it relied on data from a single informant, the majority of whom were mothers. Parents were a community sample who self-selected into the study, which may have resulted in those with lower levels of behavioural and emotional problems than a clinical population. We are also unable to assess overall participation rates as all participants self-selected into the study.

Parents were randomly assigned to a waitlist or treatment condition. In the absence of a comparison with another type of intervention or placebo condition it is difficult to distinguish genuine treatment effects. There was a high attrition rate at Time 3 (49 %). This rate may inflated the results of data analysis of treatment effects, however there were no significant differences between completers and dropouts on the outcome measures. As a result, we do not know whether all PPAP completers maintained their gains. A further point on distinguishing genuine treatment effects is that we were unable to conduct further analyses, e.g., Intention to Treat or Dropout analyses, as we do not have access to the original data set used in the study.

Overall the reliability coefficients for the outcome measures were satisfactory. However, the reliability coefficient for the SDQ Conduct Problems subscale was 0.51, which could be described as poor. As the study comprised a community based sample with relatively low rates of emotional and behaviour difficulties, it may be that the narrow range of scores reduced the reliability of this subscale.

### Implications for practice and policy

The present findings indicate that the PPAP programme is effective when delivered in community-based settings. Cunningham, Bremner, and Boyle [55] highlighted the benefits of delivering group parent-training in a community setting over a clinical setting, reporting that, in addition to offering a more cost-effective alternative to administering parent-training in a clinic setting, it also provides a service to families that is non-stigmatising, and thus more appealing, with the potential for reaching greater numbers of parents.

While parent training programmes have been shown to have a positive impact on a range of outcomes the availability of these educational programmes is limited and many parents do not receive the support they need [9]. With current media attention focusing on adolescent anti-social behaviour and taking into account the protective role of parents in preventing negative outcomes for their children, the availability and accessibility of parenting programmes at a community level needs to be addressed. Furthermore, given the high social and economic costs associated with ineffective parenting, the implementation of policies supporting education programmes, aimed at the development of positive parenting skills, should be prioritised.

Of note, is that the majority were parents of children aged 12 years. That these parents benefited from a programme aimed at adolescents may be explained by the fact that all were parents of children transitioning to secondary school, where the same demands would be placed on a cohort of students in their first year of secondary education irrespective of age. The implication for practice is that programmes need to be offered to parents on the basis of the developmental stage of their children.

### Implications for future research

As this study targeted adolescents transitioning from primary to post-primary school, the mean age of the adolescents was in the younger age-group, notably 12.34 years. In addition, the majority of the young people were girls (61 %). In future studies it would be useful to explore the effectiveness of the PPAP with older adolescents, who may have more significant behavioural problems, and with a more balanced gender group. In addition, future research could aim to explore the factors that affect parenting programme attendance, as well as responsiveness to the intervention and maintenance of treatment gains. Furthermore, it would also be important to consider the role of adolescents in parenting programmes, i.e., what role do adolescents have to play in being involved in the interventions aimed at them, which involve their parents attending parent training. It would also be valuable to consider gathering data from adolescents whose parents have attended parent training in evaluating such programmes. Of note, is that the PPAP does provide scope for facilitators to implement individual family sessions, which involve adolescents and parents. It would be useful to include data gathered at these meetings in future research on PPAP. Fathers of adolescents are also important players who need to be considered in future research. Particular thought needs to be given to engaging fathers in parenting programmes and in programme evaluation.

It would be important for researchers carrying out research in a community based sample to consider the resource implications of trying to gather follow-up from a sample of participants who may be ‘harder to reach’ by not being linked with a particular service. One suggestion from the researchers involved in the current study is to reengage participants by inviting them to follow-up programme or information sessions.

Future research would be strengthened through the inclusion of qualitative data. Qualitative research is increasingly seen as having implications for practice and its inclusion in methodology has been both called for and supported in the research literature (e.g., [56]).

## Conclusions

This study provides preliminary evidence that PPAP may be an effective model of parent-training implemented in a community-based setting. The results are timely, given that the education, health, and social services, both within Ireland and globally, are experiencing dramatic cuts in funding and resources and cost-effective interventions are now needed. Parenting adolescents is a complex, challenging and significant life task and the PPAP may be seen as an important resource for supporting parents in their vital parental role.
